# Silymarin from Milk Thistle Fruits Counteracts Selected Pathological Changes in the Lenses of Type 1 Diabetic Rats

**DOI:** 10.3390/nu14071450

**Published:** 2022-03-30

**Authors:** Weronika Borymska, Maria Zych, Sławomir Dudek, Ilona Kaczmarczyk-Sedlak

**Affiliations:** Department of Pharmacognosy and Phytochemistry, Faculty of Pharmaceutical Sciences in Sosnowiec, Medical University of Silesia, Katowice, Jagiellońska 4, 41-200 Sosnowiec, Poland; mzych@sum.edu.pl (M.Z.); sdudek@sum.edu.pl (S.D.); isedlak@sum.edu.pl (I.K.-S.)

**Keywords:** silymarin, diabetes, lenses, oxidative stress, polyol pathway, rats

## Abstract

Diabetes is a metabolic disease affecting many tissues and organs. The main etiological factor for diabetic complications is hyperglycemia and subsequent pathologies, such as oxidative stress. One of the organs susceptible to the development of diabetic complications is the eye with all of its elements, including the lens. The aim of this study was to evaluate the effect of silymarin, an extract obtained from milk thistle fruit husks, on the oxidative stress markers in the lenses of type 1 diabetic rats. The study was performed on male rats in which type 1 diabetes was induced with 60 mg/kg streptozotocin injection. Diabetic animals were treated via an intragastric tube with silymarin at 50 and 100 mg/kg doses for four weeks. Multiple oxidative stress and polyol pathway-related parameters were measured in the lenses, and auxiliary biochemical tests in the serum were conducted. Diabetes induced severe pathological changes both in the lenses and the serum, and silymarin counteracted several of them. Nevertheless, the qualitative analyses encompassing all tested parameters indicate that silymarin slightly improved the overall state of diabetic animals. Upon the obtained results, it can be concluded that silymarin reveals a faint positive effect on the lenses in type 1 diabetic rats.

## 1. Introduction

Silymarin is a flavonoid-containing mixture extracted from the seeds and fruits of the milk thistle plant—*Silybum marianum* (L.) Gaertn. It is available as dietary supplements and can be registered as a drug [1,2,3,4]. The composition of silymarin may vary depending on the plant variety, but its main constituent is flavonolignan called silybin A or silibinin. Silymarin also contains numerous additional compounds, including taxifolin-derived flavonolignans, flavonoids, and other polyphenolic substances [5,6]. Silymarin is mostly known for its hepatoprotective activity [6,7,8]. Apart from its beneficial impact on the liver, silymarin reveals positive effects on allergic rhinitis or arthritis [9], has a promising therapeutic potential in neurodegenerative disorders, such as Parkinson’s disease or Alzheimer’s disease [10,11], acts as an antioxidant [12], and can be helpful in patients with metabolic diseases, such as metabolic syndrome [13], cardiometabolic syndrome [14], or diabetes mellitus and its complications [9,15]. Clinical studies involving healthy people and various patient groups revealed that silymarin is generally a safe and well-tolerated drug with no or mild side effects [9].

Diabetes mellitus is a metabolic disorder characterized by hyperglycemia resulting from insulin deficiency, insulin resistance, or both. Although there are many types of diabetes, two of them are dominant: type 1 and type 2 diabetes [16]. In type 1 diabetes, hyperglycemia results from insulin deficiency due to the loss of pancreatic β cells [17,18]. Diabetes accompanied by long-term hyperglycemia affects multiple organs, leading to the development of diabetic complications. One of the organs susceptible to hyperglycemia is the eye. Numerous ophthalmic pathologies may develop due to diabetes, including diabetic retinopathy, diabetic choroidopathy, ocular neuropathies, glaucoma, cornea edema, and diabetic cataract [19,20]. A cataract is an ocular impairment affecting the lens, leading to its clouding and, eventually, to blindness. It is said that diabetic patients are five times more prone to cataract onset than their healthy contemporaries. What is more, cataract develops at an earlier age in people with diabetes. Cataract removal surgery is a common and safe procedure, but in diabetic patients, there is a higher risk of postsurgical complications. Many factors contribute to cataract formation in diabetic patients, including hyperglycemia and downstream pathological processes resulting from it, such as oxidative stress, glycation of macromolecules, and polyol pathway intensification [21,22]. Medicinal plant extracts, plant-derived compounds, including flavonoids, are capable of diabetic cataract prevention, which was proven in numerous in vitro and in vivo studies [23,24,25]. Silymarin, and its main component, is also described as a potential agent to mitigate cataract formation in vivo or alleviate the pathological processes in the lenses observed in the in vitro cultures. The beneficial effect of both silymarin and silibinin on the lenses was shown in an ex vivo experiment conducted on goat lenses cultured in a high glucose environment [26]. Silibinin was also demonstrated as an agent protecting rabbit lenses against oxidative stress in the in vitro study, in which the lenses were incubated in a Fenton reaction system [27]. Moreover, two in vivo tests were performed to find if silymarin has a positive effect on cataract formation: in rats with galactose-induced cataract [28] and in rabbits with the cataract induced by sodium selenite [29]. In both these studies, silymarin turned out to be an effective anticataract agent [28,29]. However, none of these reports focuses on the changes occurring in the lenses in the course of diabetes. 

Based on the available literature data, it can be hypothesized that silymarin may also positively affect the lenses in diabetic conditions. Therefore, this study aimed to investigate if the oral administration of silymarin counteracts negative alterations in the lenses of streptozotocin-induced type 1 diabetic rats. 

## 2. Materials and Methods

### 2.1. Animals, Diabetes Induction, and In Vivo Study Design

The experiment was carried out on the rat model of type 1 diabetes. For the purposes of the study, three-month-old Wistar male rats were purchased from the Centre of Experimental Medicine at the Medical University of Silesia in Katowice. The rats were divided into four experimental groups: NDM—nondiabetic control rats; T1DM—diabetic control rats, in which type 1 diabetes was induced; SIL50—type 1 diabetic rats treated with silymarin at a dose of 50 mg/kg for 28 days; SIL100—type 1 diabetic rats treated with silymarin at a dose of 100 mg/kg for 28 days. Silymarin doses and administration periods were chosen based on the literature data [30,31,32]. The specific details about room conditions are given in the “Institutional Review Board Statement” at the end of the manuscript.

Type 1 diabetes was induced in the T1DM, SIL50, and SIL100 groups of rats by a single injection of streptozotocin (STZ; Cayman Chemical, Ann Arbor, MI, USA) at a dose of 60 mg/kg [33]. STZ was prepared as a solution in a 0.1 M citric buffer (pH 4.5). In order to maintain the same conditions, the NDM group of rats was vehicle treated with the citric buffer only. The adequate volume of STZ solution or citric buffer injection was adjusted according to the current weight of each rat. The development of diabetes was then checked after two weeks: the tip of the tail was punctured, and the obtained capillary blood was used to control blood glucose level with a MicroDot glucometer equipped with test strips (Cambridge Sensor USA, Plainfield, IL, USA). For the further steps of this study, only the rats in which glucose level exceeded 200 mg/dL were chosen. The rats from the NDM and T1DM groups were given water at a volume of 1 mL/kg via an intragastric tube. The rats in the SIL50 and SIL100 groups were treated with silymarin at the doses of 50 mg/kg and 100 mg/kg, respectively. Silymarin was obtained from tablets of a commercially available OTC drug containing *Silybum marianum* fruit husk extract standardized for silymarin content. The drug tablets were pulverized and weighted, then suspended in an adequate amount of water to obtain a 50 or 100 mg/kg dose. The suspension was given by an intragastric tube at a 1 mL/kg volume. The volume of water or silymarin suspension was adjusted according to the current body weight of each animal. All rats were treated for 28 consecutive days. One day after treatment was over, the animals were euthanized by sedation with a single intraperitoneal injection of ketamine and xylazine mixture (Ketamina 10%, Biowet Puławy Sp. z o. o., Puławy, Poland and Xylapan, Vetoquinol Biowet, Gorzów Wlkp., Poland; 87.5 mg/kg of ketamine and 12.5 mg/kg of xylazine was injected), followed by the collection of the whole volume of the blood from the heart. The collected blood was then centrifuged in order to obtain the serum for biochemical analyses.

The eyeballs were extracted from the euthanized animals, and then the lenses were separated from the remaining eyeball structures. The lenses were weighted and homogenized manually with a glass homogenizer in a phosphate-buffered saline (PBS), pH 7.4 (10% *v*/*w*). The obtained homogenate was divided: one part was frozen right after homogenization (the total homogenate), while the other was centrifuged for 15 min at 10,000× *g* and +4 °C. The supernatant was collected from this centrifuged homogenate, while the residue was discarded. Both the supernatant and total homogenate were used for further biochemical analyses.

All procedures were accepted by the Local Ethics Committee in Katowice, Poland (approval nos. 36/2015 and 114/2015).

### 2.2. Assessment of Basic Serological Parameters

Glucose, fructosamine, total cholesterol, LDL-cholesterol, HDL-cholesterol, triglycerides, urea BUN, and uric acid levels, as well as the activity of aspartate transaminase (AST) and alanine transaminase (ALT), were measured with the commercially available colorimetric kits (BioSystems S.A., Barcelona, Spain). The insulin level was evaluated with an ELISA kit (Ultrasensitive Rat Insulin ELISA, Mercodia AB, Uppsala, Sweden).

### 2.3. Assessment of Protein Level in the Lenses

The protein level in the lenses was assessed using the Lowry’s method [34] using bovine serum albumin (Sigma-Aldrich, St. Louis, MO, USA) as a standard. To evaluate the total protein level, the method was performed on the total homogenate, while soluble protein was measured in the supernatant obtained after centrifugation. The ratio of total to soluble protein was calculated.

### 2.4. Assessment of Antioxidative Enzyme Activity in the Lenses and the Serum

Superoxide dismutase (SOD), catalase (CAT), and glutathione peroxidase (GPx) activities were assessed both in the lenses and the serum. For this purpose, commercially available kits were used (Cayman Chemical, Ann Arbor, MI, USA). Additionally, in the lenses, the activities of glutathione reductase (GR) and glucose-6-phosphate dehydrogenase (G6PD) were evaluated. GR activity was measured with an appropriate Cayman kit, while G6PD activity was tested with a Pointe Sci. kit (Pointe Scientific, Inc., Canton, MI, USA). The activity of these enzymes was standardized per protein level: for the lenses, the values of soluble protein were used, as the activity of these enzymes was measured in the supernatant, and the protein level in the serum was measured with BioSystems colorimetric kit.

### 2.5. Assessment of Thiol and Glutathione Levels in the Lenses

The supernatant collected from centrifuged lens homogenate was also used to evaluate the thiols and glutathione levels in the lenses. Total thiols (Total-SH) level was measured with a method presented by Ellman [35], and nonprotein thiol groups (NP–SH) were measured with a protocol proposed by Sedlak and Lindsay [36], and extinction coefficient = 13,600/M/cm was used for calculations. Protein thiol groups (P–SH) were calculated by subtracting NP–SH from Total-SH. As far as glutathione level is concerned, a commercially available kit (Cayman Chemical, Ann Arbor, MI, USA) was used. The kit makes it possible to measure total glutathione (Total GSH) and oxidized glutathione (GSSG) levels, and subtracting GSSG from Total GSH results in the value of reduced glutathione (GSH) level.

### 2.6. Assessment of Oxidative Damage Markers, Vitamin C, and Advanced Glycation End Product Level in the Lenses and the Serum

The level of advanced glycation end products (AGEs) in the lenses and the serum was measured using an OxiSelect ELISA kit (Cell Biolabs, Inc., San Diego, CA, USA). The advanced oxidation protein products (AOPP) were measured in the lens supernatant, and the serum using the Witko-Sarsat et al. method [37] with chloramine T (Sigma-Aldrich, St. Louis, MO, USA) used as a standard, and the malondialdehyde (MDA) level was measured in the serum, and in the lens total homogenate according to the method presented by Ohkawa et al. [38], and 1,1,3,3-tetraethoxypropane (Sigma-Aldrich, St. Louis, MO, USA) was used as a reference. Moreover, the level of protein carbonyls (PCG) in the lens supernatant was measured with an OxiSelect kit (Cell Biolabs, San Diego, CA, USA). Vitamin C level in the lens supernatant was evaluated according to Jagota and Dani protocol [39] with the standard curve prepared with vitamin C (L-ascorbic acid, Eurochem BGD Sp. z o.o., Tarnów, Poland).

### 2.7. Assessment of the Total Oxidative Stress Markers in the Lenses and the Serum

The supernatant obtained from the centrifugation of the lens homogenate and the serum was used in order to evaluate the following markers describing the oxidative stress: total antioxidant response (TAR), which was assayed according to the protocol presented by Erel [40], total oxidative status (TOS), which was measured as presented in other Erel’s work [41], and oxidative stress index (OSI), which was calculated as follows: OSI = TOS/(TAR × 100) [42]. Trolox ((±)-6-Hydroxy-2,5,7,8-tetramethylchromane-2-carboxylic acid; Sigma-Aldrich, St Louis, MO, USA) and hydrogen peroxide (Cayman Chemical, Ann Arbor, MI, USA) were used for standard curve preparation in the TAR and TOS methods, respectively.

### 2.8. Assessment of Polyol Pathway Markers in the Lenses

The glucose and fructose levels in the lenses were evaluated by the colorimetric BioSystems kit. Aldose reductase (AR) activity was measured according to the method presented by Patel et al. [43], and the activity of sorbitol dehydrogenase was measured with a QuantiChrom kit (BioAssay Systems, Hayward, CA, USA). An EnzyChrom kit (BioAssay Systems, Hayward, CA, USA) was used for sorbitol level measurement.

### 2.9. Apparatus and Statistical Analysis

All measurements were performed in a Tecan Infinite M200 PRO plate reader with Magellan 7.2 software (Tecan Austria, Grödig, Austria).

The results obtained from the measurements and adequate calculations were subjected to statistical analyses. As for quantitative analysis, a one-way analysis of variance (ANOVA) followed by a Fisher’s Least Significant Difference (LSD) post hoc test was used. ANOVA and the post hoc were calculated in the Statistica 13.3 software (TIBCO Software Inc., StatSoft, Kraków, Poland). The results were considered significant if *p* < 0.05. The data in the tables and the figures are presented as arithmetical mean ± standard deviation (SD). The qualitative analysis was performed using a principal component analysis (PCA). PCA was calculated in the Past 3.21 software [44] using a correlation matrix. Scores for PC1 and PC2 were subsequently subjected to MANOVA with LSD post hoc test in the Statistica 13.3 software. Moreover, hierarchical clustering heatmaps were prepared with an online MetaboAnalyst 5.0 platform. After a data update to the platform, the mode of removing missing data and the mode of data normalization were set to default settings (https://www.metaboanalyst.ca/ [45] accessed on 14 November 2021). Since the rats in the diabetic groups developed severe hyperglycemia and thus were exhausted, not all animals survived until the end of the experiment. Therefore, the final number of rats in each group from which samples were collected was as follow: NDM *n* = 9, T1DM *n* = 8, SIL50 *n* = 8, and SIL100 *n* = 9. One blood sample from the SIL50 group and one from the SIL100 group hemolyzed, and the serum collection was not possible, thus the number of the samples in serological analyses was *n* = 7 and *n* = 8, respectively. This experiment was a part of a large research project, therefore the values for the control groups (NDM and T1DM) are shared with previously published studies [46,47,48,49,50].

## 3. Results

### 3.1. Effect of Silymarin on Body Weight and Serological Parameters in Rats with Type 1 Diabetes Mellitus

**Figure 1:**
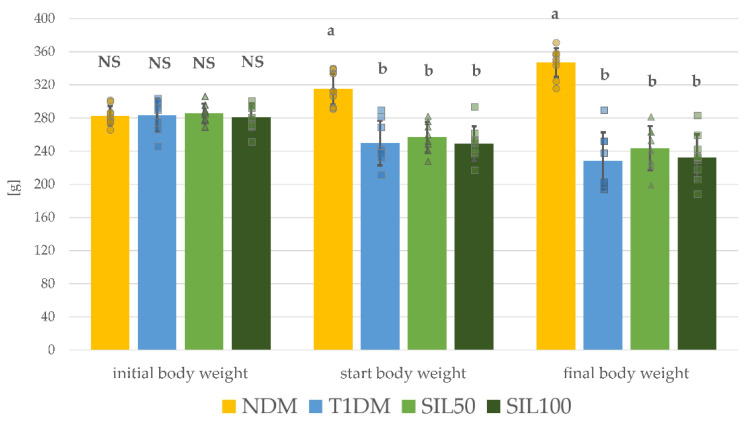
Effect of silymarin on body weight of type 1 diabetic rats: initial body weight: body weight recorded on the day of streptozotocin injection; start body weight: body weight recorded two weeks after streptozotocin injection, on a day of the beginning of water and silymarin treatment; final body weight: body weight recorded on the last day of the experiment, after 28 days of water and silymarin administration. Results in the graphs are presented as individual data points and arithmetical means ± standard deviation (*n* = 8–9). Statistical significances were evaluated with one-way ANOVA followed by Fisher’s LSD post hoc test. The letters (a, b) in the superscripts indicate statistical significances. Values presented in individual panels for each body weight recording point sharing at least one letter reveal no statistically significant differences at *p* < 0.05. NS—lack of statistical significance, NDM—nondiabetic control rats, T1DM—untreated type 1 diabetic rats, SIL50—type 1 diabetic rats treated with silymarin at a dose of 50 mg/kg, and SIL100—type 1 diabetic rats treated with silymarin at a dose of 100 mg/kg.

At the beginning of the experiment, on the day of streptozotocin (STZ) injection, the mean weight of rats (initial body weight) in each group was comparable (Figure 1), and there were no statistically significant differences among all the experimental groups of rats. Two weeks after STZ injection, when the administration of water and silymarin to the appropriate groups of rats began, the mean body weight (start body weight) of the untreated diabetic animals (T1DM), as well as rats from the silymarin-treated groups (SIL50 and SIL100), was significantly lower than in the nondiabetic control rats (the NDM group). At the end of the experiment, the final body weight of the T1DM rats remained significantly lower than in the NDM animals, and 28-day treatment with silymarin did not affect the mean final body weight of the tested animals regardless of the dose. Thus, the final body weight of the SIL50 and SIL100 rats was significantly lower than the final body weight of the NDM animals and not significantly different from the final weight of the T1DM rats. There were no significant differences in the start body weight and final body weight between the SIL50 and SIL100 groups of rats (Figure 1).

Injection of STZ at a dose of 60 mg/kg to the rats resulted in disruptions of carbohydrate and lipid metabolism (Table 1). The glucose level in the serum of the control diabetic rats (the T1DM group) was significantly elevated compared with the control nondiabetic rats (the NDM group), which were injected with the citric buffer only. The administration of silymarin for 28 days to the diabetic rats from the SIL50 and SIL100 groups at 50 and 100 mg/kg doses, respectively, did not reduce the glucose level in the serum compared to the T1DM rats. The recorded level of glucose in the serum of these animals remained significantly higher than in the serum of the NDM animals. Similar observations were made for the fructosamine level, which was significantly higher in the serum of the T1DM rats than in the serum of the NDM rats. In the SIL50 and SIL100 groups, no significant changes in this parameter were found compared to the T1DM animals. As far as the insulin level is concerned, in the serum of the T1DM, a significant decrease in this hormone level was observed compared to the NDM animals. The administration of silymarin to the SIL50 and SIL100 groups did not affect this parameter compared to the T1DM. The insulin level remained significantly lower than in the NDM rats in these groups. There were no significant differences between the administered doses with regard to all the parameters mentioned above (Table 1).

The aspartate transaminase (AST) activity did not differ among all the analyzed groups, but the activity of alanine transaminase (ALT) was significantly higher in the serum of the T1DM rats than in the serum of the NDM animals. The administration of silymarin to the diabetic animals did not decrease ALT activity in the serum of the SIL50 and SIL100 rats compared to the T1DM rats. This activity remained significantly elevated compared to the NDM rats. No differences in the ALT activity between the SIL50 and SIL100 groups were recorded (Table 1).

The induction of diabetes with streptozotocin resulted in an elevation of total cholesterol and LDL cholesterol fraction (LDL-C) levels with a simultaneous decrease in the HDL cholesterol fraction (HDL-C) level in the serum of the T1DM rats as compared with the NDM rats. The administration of silymarin to the diabetic rats resulted in a reduction of LDL-C and an elevation of HDL-C levels in both the SIL50 and SIL100 rats, but only in the SIL50 rats was the total cholesterol level significantly lower in comparison to the T1DM rats. In the SIL100 rats, the total cholesterol level was not significantly lower than in the T1DM rats, but also it was not significantly higher than in the serum of the NDM or SIL50 rats. There were no significant changes in the triglyceride levels in the serum of all analyzed rats (Table 1).

Both uric acid and urea BUN levels were significantly higher in the serum of the T1DM rats than in the serum of the NDM rats. The administration of silymarin to the diabetic rats did not affect the BUN level when compared with the T1DM animals, thus its level remained elevated in comparison with the NDM rats. The uric acid level in the serum of the SIL50 rats was significantly lower than in the serum of the T1DM and was not different from a statistical point of view from its level recorded in the NDM animals. Even though the level of uric acid in the serum of the SIL100 rats was not significantly lower compared to the T1DM rats, its level did not differ from the uric acid levels observed in the serum of the NDM and SIL50 rats (Table 1).

### 3.2. Effect of Silymarin on Protein Levels in the Lenses of Rats with Type 1 Diabetes Mellitus

There were no significant differences in the level of total or soluble proteins in the lenses of all the tested animal groups. What is more, the total protein to soluble protein ratio also was not affected by induction of diabetes or silymarin treatment (Table 2).

### 3.3. Effect of Silymarin on Antioxidative Enzymes in the Serum and the Lenses of Rats with Type 1 Diabetes Mellitus

**Figure 2:**
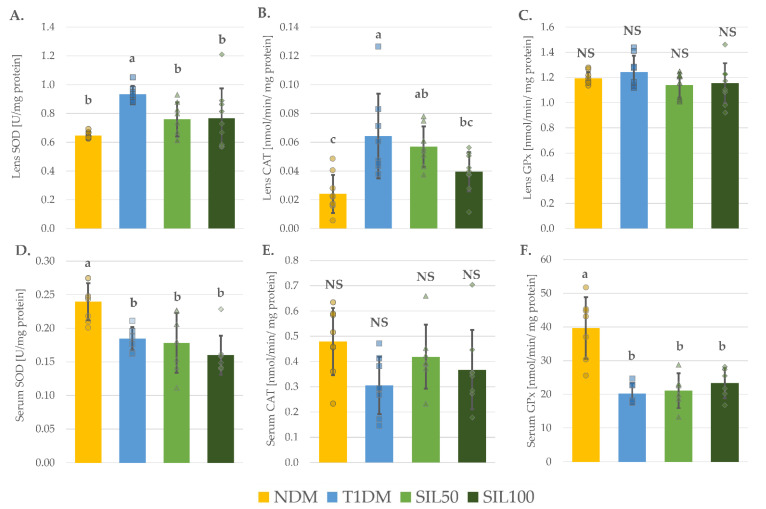
Effect of silymarin on the activity of antioxidative enzymes in the lenses and the serum of type 1 diabetic rats: (**A**) SOD activity in the lenses; (**B**) CAT activity in the lenses; (**C**) GPx activity in the lenses; (**D**) SOD activity in the serum; (**E**) CAT activity in the serum; and (**F**) GPx activity in the serum; results in the graphs are presented as individual data points and arithmetical means ± standard deviation (*n* = 8–9 for lenses assays and *n* = 7–9 for serological assays). Statistical significances were evaluated with one-way ANOVA followed by Fisher’s LSD post hoc test. The letters (a–c) in the superscripts indicate statistical significances. Values presented for each parameter in an individual panel sharing at least one letter reveal no statistically significant differences at *p* < 0.05. NS—lack of statistical significance, NDM—nondiabetic control rats, T1DM—untreated type 1 diabetic rats, SIL50—type 1 diabetic rats treated with silymarin at a dose of 50 mg/kg, SIL100—type 1 diabetic rats treated with silymarin at a dose of 100 mg/kg, SOD—superoxide dismutase, CAT—catalase, and GPx—glutathione peroxidase.

The activity of superoxide dismutase (SOD) in the lenses of control diabetic rats (the T1DM group) was significantly higher than in the lenses of the control nondiabetic NDM rats. Treating the diabetic rats with silymarin at doses of 50 and 100 mg/kg resulted in decreased SOD activity in the lenses of the SIL50 and SIL100 rats. As a result, the activity of SOD in the lenses of animals from these groups was not significantly higher than in the lenses of NDM rats. As far as the SOD activity in the serum is concerned, the induction of diabetes in the T1DM animals resulted in a decrease in the activity of this enzyme. Silymarin administration did not counteract the changes in its activity in the serum, thus the serum SOD activity in the SIL50 and SIL100 rats was significantly lower than in the serum of the NDM rats. No differences in the SOD activity in both the lenses and the serum between the two silymarin-treated groups were observed (Figure 2A,D). The catalase (CAT) activity in the lenses of the T1DM rats was significantly higher than in the lenses of the NDM rats. In the SIL50 group of rats, CAT activity was not decreased in comparison with the T1DM rats and remained significantly higher than in the lenses of the NDM rats. The administration of silymarin at a dose of 100 mg/kg to the diabetic rats resulted in a significant decrease in the CAT activity in the lenses compared to its activity in the lenses of the T1DM rats. The CAT activity in the lenses of the SIL100 rats was not higher than in the lenses of the NDM rats, but it was not significantly lower than the CAT activity in the lenses of the SIL50 rats (Figure 2B). The glutathione peroxidase (GPx) activity in the lenses of the studied animals was not altered either by diabetes induction or silymarin administration (Figure 2C). Contrary to the lenses, there were no significant differences in the CAT activity in the serum of the tested animals (Figure 2E), while the GPx activity was significantly reduced in the serum of the T1DM rats compared to the serum of the NDM rats. The administration of silymarin to the diabetic rats resulted in no changes in the serum GPx activity in comparison to the serum of the T1DM rats; thus, its activity remained significantly lower than in the serum of the NDM rats, and, as it was observed in the SOD activity, the doses did not differ among themselves (Figure 2F). As far as the glutathione reductase (GR) and glucose-6-phosphate dehydrogenase (G6PD) in the lenses were concerned, no significant differences in their activities were recorded (Table 3).

### 3.4. Effect of Silymarin on Thiols in the Lenses of Rats with Type 1 Diabetes Mellitus

**Figure 3:**
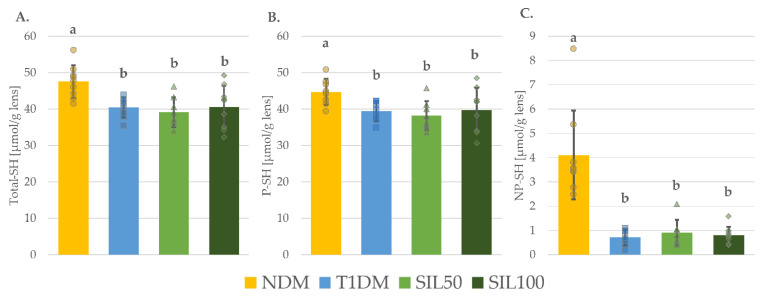
Effect of silymarin on different thiol group levels in the lenses of type 1 diabetic rats: (**A**) Total-SH level; (**B**) P–SH level; and (**C**) NP–SH level; results in the graphs are presented as individual data points and arithmetical means ± standard deviation (*n* = 8–9). Statistical significances were evaluated with one-way ANOVA followed by Fisher’s LSD post hoc test. The letters (a,b) in the superscripts indicate statistical significances. Values presented for each parameter in an individual panel sharing at least one letter reveal no statistically significant differences at *p* < 0.05. NDM—nondiabetic control rats, T1DM—untreated type 1 diabetic rats, SIL50—type 1 diabetic rats treated with silymarin at a dose of 50 mg/kg, SIL100—type 1 diabetic rats treated with silymarin at a dose of 100 mg/kg, Total-SH—total thiol groups, P–SH—protein thiol groups, and NP–SH—nonprotein thiol groups.

**Figure 4:**
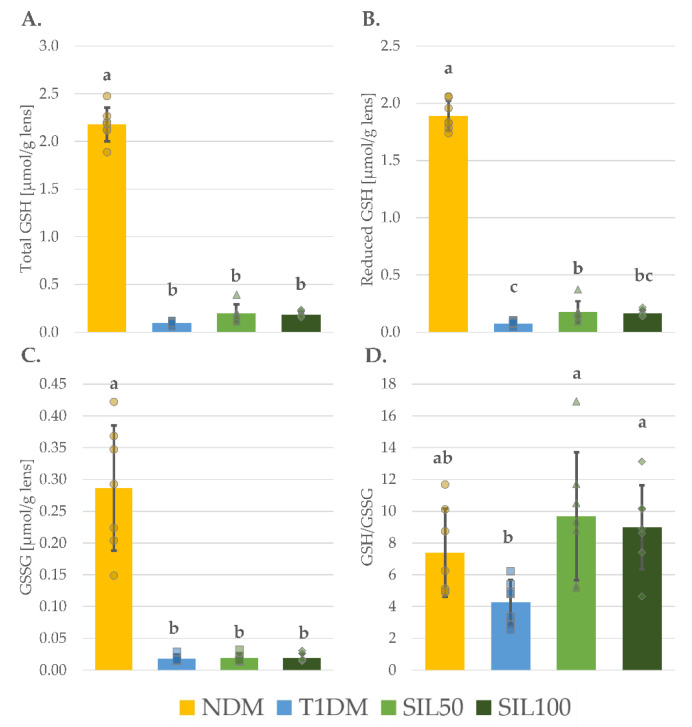
Effect of silymarin on different glutathione form levels in the lenses of type 1 diabetic rats: (**A**) Total GSH level; (**B**) Reduced GSH level; (**C**) GSSG level; and (**D**) Reduced GSH to GSSG ratio; results in the graphs are presented as individual data points and arithmetical means ± standard deviation (*n* = 8–9). Statistical significances were evaluated with one-way ANOVA followed by Fisher’s LSD post hoc test. The letters (a–c) in the superscripts indicate statistical significances. Values presented for each parameter in an individual panel sharing at least one letter reveal no statistically significant differences at *p* < 0.05. NDM—nondiabetic control rats, T1DM—untreated type 1 diabetic rats, SIL50—type 1 diabetic rats treated with silymarin at a dose of 50 mg/kg, SIL100—type 1 diabetic rats treated with silymarin at a dose of 100 mg/kg, GSH—glutathione, and GSSG—glutathione disulfide, an oxidized form of glutathione.

In the lenses of the control diabetic rats (the T1DM group), the levels of total thiol groups (total-SH), protein thiol groups (P–SH), and nonprotein thiol groups (NP–SH) were significantly reduced in comparison with their levels in the lenses of the nondiabetic control NDM animals (Figure 3A–C). Moreover, the levels of both reduced and oxidized glutathione forms (reduced GSH and GSSG, respectively) were also significantly lower in the lenses of the T1DM rats than in the lenses of the NDM rats (Figure 4B,C). The total GSH level, which represents both these forms collectively, was also significantly decreased in the lenses of the T1DM animals as compared to the NDM rats (Figure 4A). As both the reduced GSH and GSSG were lower, their ratio in the lenses of the T1DM was slightly lower than in the lenses of the NDM rats, but this decrease was statistically insignificant (Figure 4D). The administration of silymarin resulted in the changes in the level of reduced GSH only and only at a dose of 50 mg/kg; the reduced GSH level in the lenses of the SIL50 rats was significantly higher than in the lenses of the T1DM rats, but it still remained significantly lower than in the lenses of the NDM rats. In the lenses of the SIL100 rats, the reduced GSH level was slightly elevated but not enough to be significantly higher than in the lenses of the T1DM rats. Nevertheless, in the SIL100 rats, the reduced GSH level was not significantly different from the reduced GSH level observed in the lenses of the SIL50 rats, yet still significantly lower than in the lenses of the NDM rats (Figure 4B). As a result of the increase in the reduced GSH level in the lenses of the SIL50 rats, the GSH/GSSG ratio increased significantly to the GSH/GSSG ratio observed in the lenses of the T1DM rats, and it was not significantly different from the GSH/GSSG ratio recorded in the lenses of the NDM rats. Interestingly, a similar change in the GSH/GSSG ratio was also observed in the lenses of SIL100 rats, despite the fact that the observed increase in the reduced GSH level in the lenses of these rats was statistically insignificant compared to the T1DM rats (Figure 4D). The levels of total-SH, P–SH, and NP–SH, as well as of total GSH and GSSG after silymarin treatment, did not increase in comparison with their levels in the lenses of T1DM rats and remained significantly lower than in the lenses of the NDM rats (Figure 3A–C and Figure 4A,C).

### 3.5. Effect of Silymarin on Oxidative Damage and Glycation in the Lenses and the Serum of Rats with Type 1 Diabetes Mellitus

In the serum and the lenses of the type 1 diabetic rats (T1DM), there was an increase in the malondialdehyde (MDA) level as compared to the rats from the control nondiabetic group (NDM). The administration of silymarin at both doses reduced the MDA level, but only in the lenses of the tested animals. The MDA levels in the serum of the SIL50 and SIL100 rats remained unchanged compared to the serum MDA level recorded for the T1DM rats. Thus, this parameter was still elevated when compared with the serum MDA level of the NDM rats. No significant differences in the MDA levels in the serum or the lenses were noted between the two groups receiving different silymarin doses (Table 3). The opposite situation after silymarin administration was observed when advanced glycation end products (AGEs) were taken into consideration. While AGEs levels were higher in the T1DM rats than in the NDM rats both in the serum and the lenses, only in the serum was their level reduced in the SIL50 and SIL100 groups compared to the T1DM (Table 3). Advanced oxidation protein products (AOPP) level in the serum of all the tested animals did not change either after diabetes induction or after silymarin treatment. However, the AOPP level in the lenses of the T1DM rats was significantly elevated when compared to the NDM rats. Treatment with silymarin resulted in a significant decrease in this parameter to such an extent that the values for AOPP recorded in the SIL50 and SIL100 groups were significantly lower even in comparison with the AOPP values obtained in the lenses of the NDM rats (Table 3). The protein carbonyl groups (PCG) level was also elevated in the lenses of the T1DM rats when compared to the NDM rats. Only the higher dose of silymarin—100 mg/kg—reduced the PCG level to the level comparable with the level noted in the lenses of the NDM rats, and this level was significantly lower than in the lenses of the T1DM and SIL50 rats (Table 3). The vitamin C level in the lenses of the T1DM animals was significantly reduced compared to the level obtained in the lenses of the NDM rats, and silymarin treatment did not improve this parameter (Table 3).

### 3.6. Effect of Silymarin on Total Oxidative Stress Markers in the Lenses and the Serum of Rats with Type 1 Diabetes Mellitus

**Figure 5:**
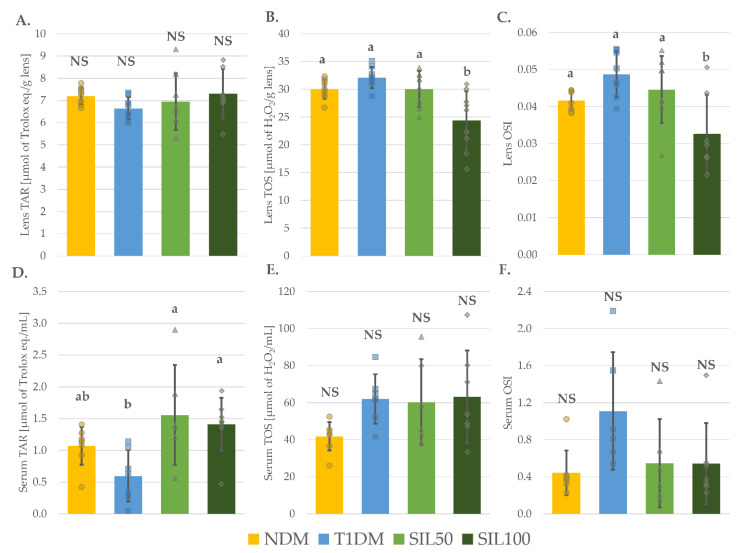
Effect of silymarin on total oxidative stress markers in the lenses and the serum type 1 diabetic rats: (**A**) Lens TAR; (**B**) Lens TOS; (**C**) Lens OSI; (**D**) Serum TAR; (**E**) Serum TOS; and (**F**) Serum OSI; results in the graphs are presented as individual data points and arithmetical means ± standard deviation (*n* = 8–9 for lenses assays and *n* = 7–9 for serological assays). Statistical significances were evaluated with one-way ANOVA followed by Fisher’s LSD post hoc test. The letters (a, b) in the superscripts indicate statistical significances. Values presented for each parameter in an individual panel sharing at least one letter reveal no statistically significant differences at *p* < 0.05. NS—lack of statistical significance, NDM—nondiabetic control rats, T1DM—untreated type 1 diabetic rats, SIL50—type 1 diabetic rats treated with silymarin at a dose of 50 mg/kg, SIL100—type 1 diabetic rats treated with silymarin at a dose of 100 mg/kg, TAR—total antioxidant response, TOS—total oxidative status, and OSI—oxidative stress index.

There were no significant changes in the total antioxidant response (TAR) in the lenses within all the tested groups. In the serum, TAR in the diabetic control T1DM animals was not different from the TAR value obtained in the serum of the control nondiabetic NDM rats. The administration of silymarin to the rats from the groups SIL50 and SIL100 elevated the serum TAR values compared to their values recorded in the serum of the T1DM animals, and these values did not differ significantly from the TAR serum value observed in the NDM rats (Figure 5A,D). On the other hand, total oxidative status (TOS) and oxidative stress index (OSI) values in the serum of the tested animals were not significantly different among all the tested groups (Figure 5E,F). The TOS and OSI values obtained in the lenses were not affected by diabetes induction or silymarin treatment at a dose of 50 mg/kg. Only the silymarin dose of 100 mg/kg reduced TOS and OSI values in the lenses of diabetic animals—these values in the lenses of the SIL100 rats were significantly lower than in the lenses of the NDM, T1DM, and SIL50 groups (Figure 5B,C).

### 3.7. Effect of Silymarin on Polyol Pathway in the Lenses of Rats with Type 1 Diabetes Mellitus

After diabetes induction, the levels of all sugars from the polyol pathway, i.e., glucose, sorbitol, and fructose, were elevated in the lenses of the T1DM rats when compared to the lenses of the nondiabetic NDM rats. Treatment with silymarin at the doses of 50 and 100 mg/kg did not affect glucose, sorbitol, or fructose levels in the lenses compared to their levels recorded in the lenses of the T1DM rats. Thus, these parameters remained significantly higher than in the lenses of the NDM rats. While in the case of the glucose level in the lenses, there were no differences between the used doses of silymarin; the differences between the SIL50 and SIL100 groups regarding the two other sugars were noted. The sorbitol level in the lenses of SIL50 animals was significantly lower than in the lenses of the SIL100 rats, while the fructose level was lower in the lenses of the SIL100 rats than in the lenses of the SIL50 rats (Table 4). Aldose reductase (AR) activity was significantly higher in the lenses of the T1DM rats, and silymarin treatment did not counteract this change. Sorbitol dehydrogenase (SDH) activity was also greater in the lenses of T1DM animals than in the lenses of the NDM rats. The SDH activity in the SIL50 rats was not significantly different from its activity in the lenses of T1DM or NDM rats. In contrast, in the lenses of the SIL100 rats, the SDH activity was higher than in the lenses of the NDM animals and was even greater than in the lenses of the T1DM and SIL50 animals (Table 4).

### 3.8. General Effect of Silymarin on the Serum and the Lenses of Rats with Type 1 Diabetes Mellitus

**Figure 6:**
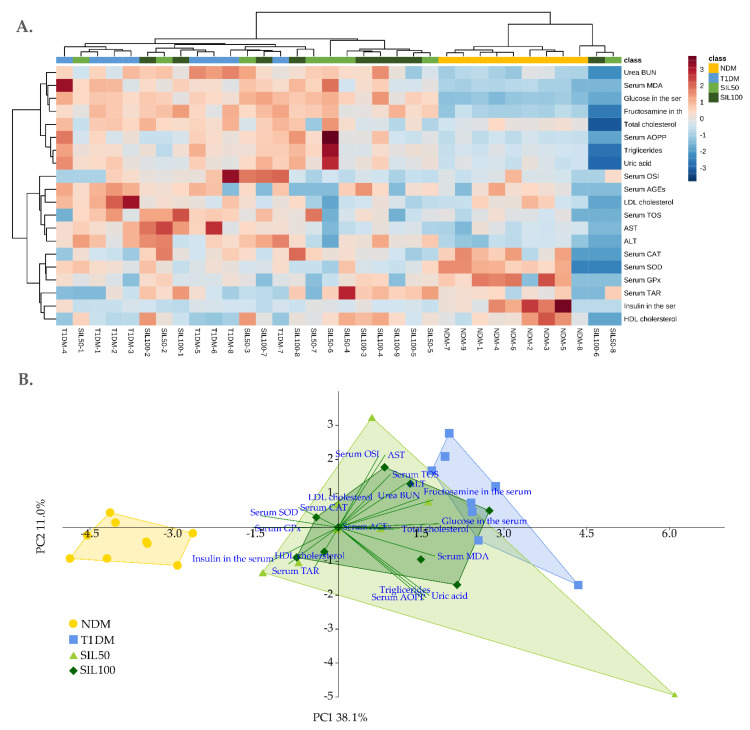
(**A**) Hierarchical clustering heatmap and (**B**) principal component analysis biplot of parameters measured in the serum of type 1 diabetic rats. NDM—nondiabetic control rats, T1DM—untreated type 1 diabetic rats, SIL50—type 1 diabetic rats treated with silymarin at a dose of 50 mg/kg, SIL100—type 1 diabetic rats treated with silymarin at a dose of 100 mg/kg, AOPP—advanced oxidation protein products, AGEs—advanced glycation end products, MDA—malondialdehyde, SOD—superoxide dismutase, CAT—catalase, GPx—glutathione peroxidase, LDL—low-density lipoprotein, HDL—high-density lipoprotein, TAR—total antioxidant response, TOS—total oxidative status, and OSI—oxidative stress index.

As seen in the hierarchical clustering heatmap from the serum parameters (Figure 6A), there was a visible distinction between the cluster formed by the NDM class and the cluster formed by the samples obtained from other groups. There was no visible cluster formation within the groups with STZ-induced diabetes—individual samples for T1DM, SIL50, and SIL100 rats were interlaced with each other; however many samples from the SIL50 and SIL100 were placed nearer the cluster formed by the samples from the NDM group than the T1DM samples. Moreover, the biochemical features were also clustered. For instance, there were features whose values were lower in the cluster formed by the NDM samples and two individual samples from the SIL50 and SIL100 groups and were higher in the samples from the groups with induced type 1 diabetes. The clustering observed in the heatmap was also visible in the principal analysis (PCA) plot (Figure 6B). The cluster formed by the NDM group was strongly separated from all other clusters with regard to the principal component 1 (PC1) axis, which explained over 38% of the total variability. Although the plot convex hulls for clusters formed by the SIL50 and SIL100 groups overlapped with the T1DM cluster, the MANOVA analysis revealed statistically significant variation along the PC1 axis, as SIL50 and SIL100 clusters were separated from the T1DM cluster and shifted towards the NDM cluster (Table 5), which confirmed the hierarchical clustering. There was no significant separation with regard to the PC2 when serum biochemical parameters were considered. The parameters that mostly affected the separation along the PC1 were glucose level, total cholesterol level, fructosamine level, and MDA level (their values were higher in the diabetic groups), and the insulin level and SOD and GPx activities – their values were higher in the serum of the NDM rats (Figure 6B). These values, among others, were also responsible for clustering presented in the heatmap (Figure 6A).

**Figure 7:**
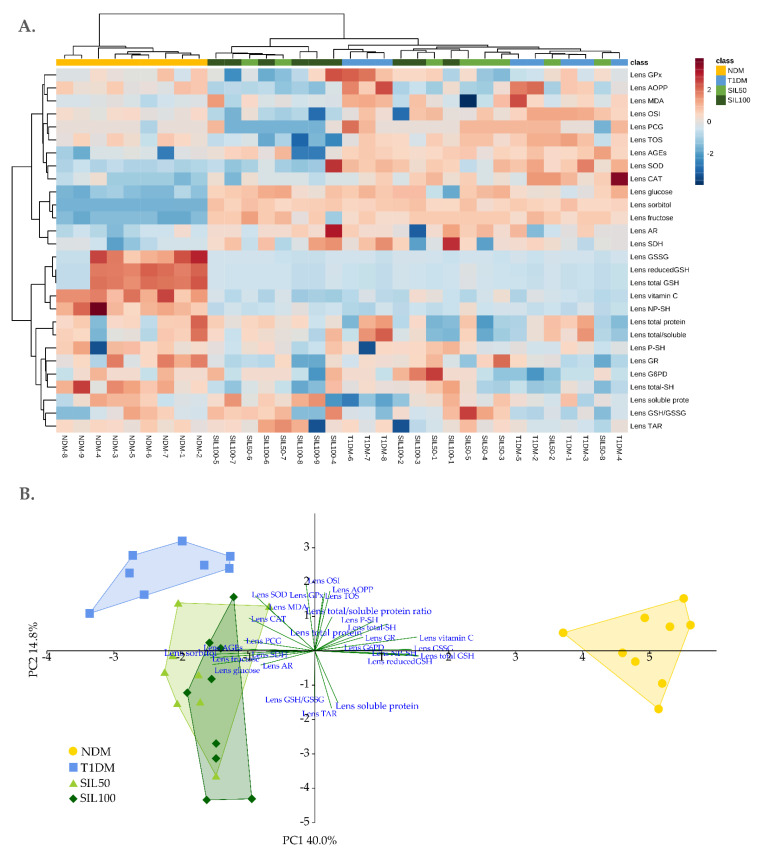
(**A**) Hierarchical clustering heatmap and (**B**) principal component analysis biplot of parameters measured in the lenses of type 1 diabetic rats. NDM—nondiabetic control rats, T1DM—untreated type 1 diabetic rats, SIL50—type 1 diabetic rats treated with silymarin at a dose of 50 mg/kg, SIL100—type 1 diabetic rats treated with silymarin at a dose of 100 mg/kg, AOPP—advanced oxidation protein products, AGEs—advanced glycation end products, MDA—malondialdehyde, PCG—protein carbonyls, GSH—glutathione, GSSG—glutathione disulfide, Total-SH—total thiol groups, P–SH—protein thiol groups, NP–SH—nonprotein thiol groups, SOD—superoxide dismutase, CAT—catalase, GPx—glutathione peroxidase, GR—glutathione reductase, G6PG—glucose-6-phosphate dehydrogenase, AR—aldose reductase, SDH—sorbitol dehydrogenase, TAR—total antioxidant response, TOS—total oxidative status, and OSI—oxidative stress index.

A similar situation was observed for the lenses. The hierarchical clustering heatmap prepared for the lenses revealed a distinctively separated cluster formed by the samples originating from the NDM group. As for the other samples—there was an overriding cluster for all diabetic groups—with one secondary cluster formed for several samples from the SIL50 and SIL100 groups (with a predominance of samples from the SIL100 group). As for the second subcluster formed with interwoven samples from the T1DM group and remaining SIL50 and SIL100 rats, there were no other pronounced clusters formed (Figure 7A). As for the feature clustering, two main branches were observed. The upper supercluster was divided into two distinctive subclusters, and the lower supercluster was more fragmented. In the upper part of the heatmap, there were parameters whose lower values were connected with the NDM cluster and the higher values with diabetes. In the bottom part of this heatmap, features with higher values in the NDM class were clustered. It can be clearly seen that features connected with the polyol pathway were clustered together and that their values were remarkably higher in the supreme cluster formed by the samples from all diabetic groups than in the NDM cluster. In contrast, a strongly pronounced cluster formed by all glutathione forms, vitamin C, and NP–SH showed high values for the NDM-formed cluster and low values for the diabetic group-formed cluster.

**Table 5:** Principal component analysis of biochemical parameters measured in the serum and the lenses of type 1 diabetic rats.

Parameter/Group	NDM	T1DM	SIL50	SIL100
**Serum**				
PC 1	−3.81 ± 0.74 ^c^	2.52 ± 0.82 ^a^	0.87 ± 2.30 ^b^	0.79 ± 1.22 ^b^
PC 2	−0.41 ± 0.52 ^NS^	0.85 ± 1.42 ^NS^	−0.34 ± 2.33 ^NS^	−0.05 ± 1.13 ^NS^
**Lenses**				
PC 1	4.97 ± 0.58 ^a^	−2.19 ± 0.77 ^c^	−1.76 ± 0.50 ^bc^	−1.46 ± 0.27 ^b^
PC 2	0.16 ± 1.01 ^b^	2.33 ± 0.68 ^a^	−0.68 ± 1.63 ^bc^	−1.63 ± 2.10 ^c^

Results in the table are presented as arithmetical means ± standard deviation. Statistical significances were evaluated with one-way ANOVA followed by Fisher’s LSD post hoc test. The letters (a–c) in the superscripts indicate statistical significances. Values presented for each parameter in individual rows sharing at least one letter reveal no statistically significant differences at *p* < 0.05. NS—lack of statistical significance, NDM—nondiabetic control rats, T1DM—untreated type 1 diabetic rats, SIL50—type 1 diabetic rats treated with silymarin at a dose of 50 mg/kg, SIL100—type 1 diabetic rats treated with silymarin at a dose of 100 mg/kg, PC 1—principal component 1, and PC 2—principal component 2.

The separation of the group clusters was also visible in the PCA plot (Figure 7B). The cluster formed by the NDM group, which was located at the right part of the plot, but in the central part of the PC2, separated strongly from all other clusters along the PC1 and from the cluster formed by T1DM and SIL100 along the PC2 (Table 5).

There was also a distinct separation of the SIL50 and SIL100 clusters from the T1DM cluster along the PC2, but as far as the PC1 was concerned, only the cluster formed by the SIL100 was separated from the T1DM cluster in a significant manner and thus was placed in between the T1DM and NDM clusters, but still closer to the T1DM than the NDM cluster. In addition, the SIL100 cluster was significantly separated from the NDM cluster along the PC2, as it was located nearer the bottom part of the plot. It should be highlighted that there were no significant differences between the SIL50 and SIL100 groups with regards to separation along both the PC1 and PC2 (Table 5). The separation along the PC1 resulted from the AGEs and polyol pathway elements correlating with the clusters located to the left of the plot (diabetic groups) and all glutathione forms, vitamin C, and NP–SH, which correlated with the NDM cluster. The separation along the PC2 was affected mainly by OSI, AOPP, GPx, and TOS (correlation with upper clusters) and TAR, soluble protein, and the GSH/GSSG ratio (bottom clusters). The PC1 explained 40.0%, while the PC2 explained 14.8% of total variability (Figure 7B). The features responsible for separation along the PC1 overlapped with two strongly pronounced feature clusters visible in the heatmap (Figure 7A).

## 4. Discussion

The lens is a main refractive element of the eye, which enables focusing the light on the retina [51]. It may fulfill its function due to crystallins, which are highly specialized proteins responsible for transparency and light refraction. Any modifications in these proteins, their aggregation, or precipitation lead to cataract formation—a vision impairment resulting from lens clouding. Many factors contribute to cataract formation. Among them, aging is one of the most common causes, but it may also develop in children or be a secondary result of eye trauma, environmental factors, malnutrition, or other diseases. One such disease triggering cataract onset is diabetes [52,53,54]. In our study, we used a rat model in which type 1 diabetes was induced. For this purpose, a single injection of streptozotocin (STZ) at a high dose was used [33]. The development of type 1 diabetes in the tested animals resulted in rapid weight loss, which is one of the classic symptoms of hyperglycemia connected with type 1 diabetes [17]. Indeed, there was an extreme elevation of serum glucose level and a decrease in insulin level in all the STZ-treated groups of rats. The administration of silymarin did not affect these glycemia-related parameters. Silymarin is reported to reduce glucose levels in diabetic animals and diabetic patients involved in clinical trials [55,56,57,58,59], but not all reports confirm the ability of silymarin to normalize distorted glycemia. There are studies in which results were consistent with our observations—treatment with silymarin did not affect the elevated glucose level of the diabetic animals [30,31,60,61]. The conditions of these aforementioned studies were as follows: the induction of diabetes with a high dose of STZ, the doses, and the duration of silymarin administration were similar to the experimental design in our study [30,31]; the dose of silymarin was higher, and the time of administration was even longer than in our research [60] or type 1 diabetes was induced with alloxan, but the duration of treatment and silymarin doses were comparable to ours [61]. The lack of the silymarin effect on the hyperglycemia was not a just one-time situation observed only at the end of the experiment. Regardless of the dose, silymarin administration did not induce any earlier changes in glycemia, as the fructosamine level, a parameter reflecting the average glucose level over the preceding 2–3 weeks [62], was not decreased when compared to untreated diabetic rats. This can be related to the fact that in the study conducted on rats with alloxan-induced diabetes, the authors noted that insulin production in the pancreas was not elevated after silymarin treatment [61].

During diabetes, not only metabolism of carbohydrates is distorted. Diabetic patients also suffer from lipid disorders, which may result in further complications [63]. In the diabetic rats in our study, similar to other studies conducted on type 1 diabetic rats [64,65], an alteration in the lipid profile was observed. As expected, silymarin, being a hepatoprotective drug, improved the distorted lipid profile, which is consistent with other studies conducted in rats with type 1 diabetes [66,67,68], type 2 diabetes [69], and even with clinical trials with diabetic patients [70,71,72].

The lens is an insulin-independent organ. In normoglycemic conditions, glucose in the lens is metabolized via glycolysis and, to a lesser extent, by pentose phosphate and polyol pathways [73,74]. When the glucose level is high, glycolysis becomes insufficient, and the polyol pathway (also known as the sorbitol pathway) takes the main lead [73]. This phenomenon was observed in our study. The elevated level of the serological glucose resulted in an elevated glucose level in the lenses. This led to the accelerated activity of aldose reductase, an enzyme converting glucose to sorbitol [75], higher concentrations of sorbitol and, consequently, fructose. The administration of silymarin did not inhibit the polyol route. These results stand in contrast to the results presented by Patil et al. [26]. The authors examined the effect of several substances ex vivo, including silymarin and its main component—silibinin. The aldose reductase activity was inhibited after treatment with both these compounds [26]. Nevertheless, it should be highlighted that the mentioned study was performed ex vivo, where the lenses were directly exposed to the tested substances. In contrast, our study was conducted in vivo, and silymarin was not administered topically directly to the lenses but orally. The oral administration of drugs, including silymarin, is connected with drug metabolism and the formation of conjugates with sugars or proteins [76,77,78]; thus, its effect on a target organ may be different from this observed ex vivo.

It is known that persistent hyperglycemia induces oxidative stress, also in the eye [79]. In our study, we observed oxidative stress in the serum and the lenses of diabetic animals: the activity of antioxidative enzymes was distorted, and oxidative damage to the macromolecules occurred. The superoxide dismutase (SOD) activity in the serum was decreased, while in the lenses, it increased. In addition, the response of other tested enzymes, such as catalase (CAT) and glutathione peroxidase (GPx), differed depending on the origin of the tested sample. Such differences in enzyme reaction can result from many factors, including the cellular context or reactive oxygen species concentration, to which a tissue or an organ is currently exposed [80]. A decreased activity of antioxidative enzymes during diabetes observed in the case of the serum is consistent with other studies performed on rat models [64,65,81] and in patients with type 1 diabetes [82]. Moreover, in the serum of the diabetic rats, an increased level of malondialdehyde, a marker of oxidative damage of lipids, was observed. This is also consistent with previous reports [65,81]. Interestingly, the administration of silymarin did not counteract the negative impact of diabetes on oxidative stress markers measured in the serum of diabetic rats. Nevertheless, silymarin does not always prevent diabetes-induced alterations in the activity of oxidative enzymes. For instance, Vessal et al. [31] examined the effect of silymarin administration to diabetic rats on oxidative stress markers in the kidney. The authors observed that silymarin administered at a dose of 100 mg/kg for four weeks did not counteract negative changes in the oxidative enzyme activity [31]. On the other hand, silymarin administration to diabetic animals resulted in the reduction of the antioxidative enzyme activity in the lenses, which was elevated in the nontreated diabetic animals. Enhanced activity of these enzymes in the lenses in diabetic conditions was previously reported by other authors [83,84]. Such an increased activity can be a preventive measure in the lens against the development of oxidative damage. SOD is an enzyme that dismutates two superoxide anions into oxygen and hydrogen peroxide, which can be further decomposed by CAT into water and oxygen or by GPx utilizing glutathione (GSH) as a substrate [85]. As far as SOD in the lenses was concerned, both silymarin doses reduced its activity, but CAT activity was reduced only after treatment with the higher dose of the tested substance. The more potent antioxidative activity of the higher silymarin dose was also observed in the parameters describing total oxidative status (TOS) and oxidative stress index (OSI) in the lenses—the values for these parameters were significantly lower than in all other groups of rats, even the nondiabetic animals.

Lens macromolecules are susceptible to oxidative damage, which may affect its transparency [86]. Therefore, we examined the effect of silymarin on the markers connected with protein and lipid oxidation. Diabetes induced unfavorable changes in the lenticular lipids and proteins, leading to an increase in the following parameters: protein carbonyl groups (PCG) and advanced oxidation protein products (AOPP) depicting oxidative damage to proteins and malondialdehyde (MDA), a marker of lipid oxidation. Similar observations were made in other experiments carried out in rat diabetic models [87,88,89,90]. Silymarin at both doses counteracted the changes in the MDA and AOPP, but only the higher dose, 100 mg/kg, resulted in a decrease of the PCG level in the lenses of diabetic rats. Our previous in vivo studies reported that other flavonoids, such as naringenin or chrysin, also reduced levels of the markers connected with the oxidative damage of these macromolecules in the lenses [47,50]. Research conducted by other authors also confirms our observation. Silymarin and its main component, silibinin, inhibited protein carbonyl groups formation in the goat lenses when examined in the ex vivo conditions [26] and in the in vivo model of galactose-induced cataract silymarin reduced the MDA level in the lenses of the rats [28].

Interestingly, although in the lenses of diabetic rats the elevated levels of protein oxidation markers were noted, the level of protein, both total and soluble, did not change significantly. Glutathione (GSH) is a crucial nonenzymatic antioxidant within the lens, assuring redox homeostasis [91]. It is a cofactor for GPx, and it also protects proteins from oxidation by S-glutathionylation [85,92,93]. The protein S-glutathionylation is a reversible process in which free protein thiol groups bind with GSH, for instant, through thiol/disulfide exchange between proteins and oxidized glutathione (GSSG) or direct interaction between partially oxidized protein thiols and GSH [92]. In our study, we observed that the levels of all forms of glutathione were significantly depleted in the lenses. What is more, the levels of total thiols, nonprotein thiols, and protein thiols were also significantly lower than in the lenses of nondiabetic rats. It should also be highlighted that the activity of the enzymes taking part in the glutathione turnover was not altered in the lenses of the diabetic rats. In oxidative stress conditions, the pool of free GSH is reduced in the lenses, as it is oxidized to GSSG by glutathione peroxidase (GPx), and the amount of H_2_O_2_ produced due to SOD activity is reduced. Together with the diminishing GSH amount in the lenses, the level of S-thiolated proteins forming a glutathione-protein heterodimer (PSSG) increases. Proteins can also be S-thiolated by other nonprotein thiols, such as free cysteine or γ-glutamylcysteine forming a protein-S-S-cysteine (PSSC) or protein-S-S-γ-glutamylcysteine (PSSGC) heterodimers, respectively [94]. We presumed that in the case of the lenses examined in our study, the glutathione and other nonprotein thiols served as direct protein protective agents. These compounds blocked the protein thiol groups by S-thiolation, protecting them from cross-linking and oxidation, thus from precipitation. This is why the soluble protein content and its ratio to total protein did not change. In order to prove it, further examinations are needed in the future, including the evaluation of the levels of PSSG, PSSC, and PSSGC.

Silymarin administration at a dose of 50 mg/kg resulted in a slight increase in the GSH level as compared to the lenses of diabetic, nontreated animals. But when this level was compared to the GSH level noted in the lenses of nondiabetic rats, it was still extremely reduced, as can be clearly seen both in the Figure 4 and the heatmap presented in the Figure 7B. The authors who analyzed the effect of silymarin on the lenses of rats with galactose-induced cataract also observed a significant increase in the GSH level. Still, silymarin did not restore this level to the values observed in the lenses of the control rats [28]. It is noteworthy that total GSH, as well as GSSG levels and the levels of all forms of thiol groups: total, protein, and nonprotein remained significantly depleted after the treatment of silymarin, regardless of the dose. The GSH/GSSG ratio is considered a good marker of oxidative status [95]. Even though the GSH/GSSG ratio in the lenses of the rats administered with silymarin was higher than in the lenses of the diabetic control rats, it cannot be concluded that silymarin beneficially affected the glutathione content in these lenses, as the observed levels of GSH and GSSG were still markedly reduced as compared to their levels noted in the nondiabetic animals.

An enhanced polyol pathway results in the overgeneration of advanced glycation end products (AGEs) in the lenses [96]. In our study, in the diabetic lenses, this pathway was strongly active, and the level of AGEs in the lenses was significantly elevated. As we noted above, silymarin administration did not suppress this pathway. As a result, AGEs generation, even after silymarin treatment, was not inhibited, and its level did not decrease in the lenses of silymarin-treated animals. AGEs are compounds that can escalate oxidative stress, generate oxidative damage, induce osmotic stress, and promote protein cross-linking [97,98,99]. This suggests that the lens proteins, despite the fact that silymarin was administered, were still exposed to the risk of damage and needed to be protected from oxidation or structural changes by GSH. Moreover, as AGEs contribute to oxidative stress intensification, and their level was not reduced in the lenses of diabetic animals, even after silymarin treatment, vitamin C, another low molecular endogenous antioxidant occurring in rats [100], was significantly reduced. Presumably, it was also used to counteract AGEs-generated oxidative stress. The ex vivo test conducted on the goat lenses cultured in the high-glucose environment revealed that silymarin is a rather moderate inhibitor of AGEs formation [26]. On the other hand, Wu et al., who examined the effect of silymarin supplemented with a diet on the AGEs formation in the plasma of diabetic rats, reported that this substance inhibited AGEs formation in the plasma, and it especially lowered the level of glycated albumin [101]. We also reported an antiglycating effect of silymarin in the serum of the diabetic rats—both the doses of silymarin reduced AGEs levels in the serum of type 1 diabetic rats.

Additionally, to evaluate the overall effect of silymarin on the serological and lens parameters, we performed statistical analyses encompassing all parameters at one time. Hierarchical clustering heatmaps make it possible to present the data matrix graphically in the form of ordered clusters and help identify groups of correlated samples. Colors used in such heatmaps represent standardized data values. Moreover, because data density is high in cluster heatmaps, it is possible to present large amounts of information compacted into a small space [102]. We observed that in the heatmap prepared for the serum, samples from the nondiabetic control rats fused in a uniform cluster, while other samples from all diabetic groups (except for two SIL50 and SIL100 samples) formed a second large cluster. This suggests that the effect of diabetes itself is very strong. This also finds confirmation in the principal component analysis (PCA). In the biplot, the clusters formed by the diabetic groups were placed together and were definitely separated from the NDM cluster. In another study performed in the rats with type 2 diabetes, a similar pattern was observed—diabetic groups merged together and separated from the nondiabetic cluster [103]. Moreover, in a different study on rats with type 1 diabetes and estrogen deficiency, a PCA revealed that the effect of estrogen deficiency was negligible and was clearly dominated by the diabetes effect [104]. Contrary to ANOVA, which makes it possible to compare every single parameter according to quantitative differences, PCA is a qualitative analysis that provides information on the significance of all the measured parameters on total observed variance, highlighting these which affect this variability the most, even if they are not significant in quantitative comparisons [104,105]. In the case of the serum, the most important parameters affecting the total variance and cluster formation were glucose and fructosamine, and then MDA, whose high levels were correlated with groups of rats with type 1 diabetes. These features also determined the separation of clusters formed by groups of rats in which type 2 diabetes was induced with a high fat diet and streptozotocin [103]. When all the parameters measured in the serum were taken into consideration together, and their qualitative effect was analyzed in the PCA, it turned out that silymarin administration improved the overall condition of the rats, contrary to the ANOVA results from individual parameters. Nevertheless, the tested substance did not restore the animals’ conditions to such observed in nondiabetic rats. It is also visible in the heatmap, where many samples from SIL50 and SIL100 classes formed a subcluster located nearer the NDM cluster but were still grouped in a large cluster together with T1DM samples.

As far as the lenses are concerned, the hierarchical clustering heatmap, similar to the serum, revealed a clearly pronounced cluster formed by the NDM group. The rest of the samples were grouped together, and then subclusters were formed. Other information that can be read from this heatmap is that several features assembled in clusters: parameters connected with polyol pathway and levels of all glutathione forms, vitamin C, and nonprotein thiols. These were also the main features deciding the separation along the PC1 visible in the PCA biplot for the lenses. Also, a separation along the PC2 was noted, but different parameters determined it. It should be highlighted that although according to MANOVA, the silymarin doses did not differ between themselves, the administration of the higher dose, 100 mg/kg, resulted in a significant separation of the SIL100 cluster from the T1DM cluster along the PC1. Interestingly, the separation from the T1DM with regard to the PC2 was observed for both silymarin clusters, and the SIL100 cluster also separated significantly from the NDM cluster. The clusters formed by the samples from silymarin-treated groups of animals were located lower in the plot than the T1DM cluster, and the SIL100 even lower than the NDM cluster. We observed a similar separation pattern along the PC2 earlier, examining the effect of two other flavonoids: naringenin and chrysin [47,50]. In both these cases, clusters formed by the samples originated from flavonoid-treated diabetic rats separated along the PC2 toward the opposite part of the plot than the clusters formed by groups of rats that were not administered with the tested substances. This suggests that the effect of flavonoids on the lenses is different than their effect on the serum, as we did not note separation along the PC2 in the PCA biplot for the serum. There could be many factors affecting the response of different organs or tissues to the action of flavonoids, including their metabolism, route of transport, and bioavailability of these compounds in the targeted organ. There are many studies on the interaction of flavonoids with the serum (especially binding to albumins) [106,107,108], but with regard to the bioavailability and transport of flavonoids to the lenses, the information is scarce. Only few studies have been conducted so far, and a hypothetical mechanism was proposed for quercetin [23], but exact mechanisms are yet to be discovered.

## 5. Conclusions

Our study aimed to investigate the effect of silymarin on diabetes-induced pathological changes in the lenses of rats at the biochemical level. Diabetes, which was induced with streptozotocin, was confirmed by blood glucose level measurements and caused disturbances in oxidative, carbohydrate, and lipid homeostasis in the serum. As expected, silymarin improved the altered parameters connected with lipid metabolism in the serum, but it cannot be regarded as an antihyperglycemic drug in the course of type 1 diabetes. As a result of diabetes, severe oxidative stress and osmotic stress-related pathologies occurred within the eye lens of the rats. Our study revealed that based on the quantitative analyses performed for every single parameter separately, silymarin beneficially affected only several of them. Notably, only parameters connected with oxidative stress were improved, but no effect on the polyol pathway was noted. Nevertheless, when the general picture was evaluated based on the qualitative multivariate analyses, it turned out that silymarin improved the overall condition of the lenses in the diabetic rats, despite the lack of observed quantitative effect in individual parameters. This leads to a conclusion that silymarin affects positively the lenses of diabetic rats at a biochemical level, but the effect is delicate. In order to explain the mechanisms underlying these changes, molecular studies are necessary. Hence, more tests in more complex experimental set-ups are needed, and our research points out the possible direction for future investigations.

## Figures and Tables

**Table 1:** Effect of silymarin on serological parameters in type 1 diabetic rats.

Parameter/Group	NDM	T1DM	SIL50	SIL100
Glucose [mg/dL]	141.4 ± 33.0 ^b^	641.8 ± 80.9 ^a^	661.8 ± 75.2 ^a^	631.3 ± 149.6 ^a^
Insulin [µg/L]	0.434 ± 0.272 ^a^	0.089 ± 0.053 ^b^	0.069 ± 0.041 ^b^	0.083 ± 0.035 ^b^
Fructosamine [µmol/L]	281.9 ± 29.5 ^b^	495.6 ± 65.2 ^a^	475.6 ± 58.4 ^a^	496.2 ± 65.0 ^a^
AST [U/L]	16.50 ± 4.55 ^NS^	30.22 ± 12.61 ^NS^	25.68 ± 16.59 ^NS^	26.03 ± 11.74 ^NS^
ALT [U/L]	4.33 ± 1.67 ^b^	9.86 ± 2.78 ^a^	10.50 ± 3.49 ^a^	9.76 ± 3.05 ^a^
Total cholesterol [mg/dL]	84.3 ± 5.8 ^b^	101.5 ± 6.3 ^a^	82.9 ± 20.4 ^b^	89.7 ± 13.8 ^ab^
HDL-C [mg/dL]	48.7 ± 18.5 ^a^	19.1 ± 5.3 ^b^	47.9 ± 11.5 ^a^	45.5 ± 14.6 ^a^
LDL-C [mg/dL]	9.15 ± 2.76 ^b^	13.67 ± 5.48 ^a^	8.15 ± 2.66 ^b^	8.31 ± 1.97 ^b^
Triglycerides [mg/dL]	74.7 ± 3.5 ^NS^	96.9 ± 13.2 ^NS^	96.2 ± 33.6 ^NS^	91.1 ± 10.0 ^NS^
Uric acid [mg/dL]	5.30 ± 0.35 ^b^	6.72 ± 0.90 ^a^	6.67 ± 1.76 ^b^	6.33 ± 0.98 ^ab^
Urea BUN [mg/dL]	36.9 ± 7.8 ^b^	69.6 ± 15.6 ^a^	62.2 ± 13.0 ^a^	58.1 ± 16.1 ^a^

Results in the table are presented as arithmetical means ± standard deviation (*n* = 7–9). Statistical significances were evaluated with one-way ANOVA followed by Fisher’s LSD post hoc test. The letters (a, b) in the superscripts indicate statistical significances. Values presented for each parameter in individual rows sharing at least one letter reveal no statistically significant differences at *p* < 0.05. NS—lack of statistical significance, NDM—nondiabetic control rats, T1DM—untreated type 1 diabetic rats, SIL50—type 1 diabetic rats treated with silymarin at a dose of 50 mg/kg, SIL100—type 1 diabetic rats treated with silymarin at a dose of 100 mg/kg, AST—aspartate transaminase, ALT—alanine transaminase, HDL-C—high-density lipoprotein fraction of cholesterol, and LDL-C—low-density lipoprotein fraction of cholesterol.

**Table 2:** Effect of silymarin on protein in the lenses of type 1 diabetic rats.

Parameter/Group	NDM	T1DM	SIL50	SIL100
Total protein [mg/g]	563.2 ± 99.7 ^NS^	536.4 ± 123.5 ^NS^	487.3 ± 112.1 ^NS^	521.6 ± 69.9 ^NS^
Soluble protein [mg/g]	443.8 ± 25.2 ^NS^	408.5 ± 39.2 ^NS^	438.7 ± 11.8 ^NS^	446.0 ± 43.3 ^NS^
Total/soluble protein ratio	1.28 ± 0.27 ^NS^	1.32 ± 0.34 ^NS^	1.11 ± 0.27 ^NS^	1.18 ± 0.20 ^NS^

Results in the table are presented as arithmetical means ± standard deviation (*n* = 8–9). Statistical significances were evaluated with one-way ANOVA followed by Fisher’s LSD post hoc test. Values presented for each parameter in individual rows sharing at least one letter reveal no statistically significant differences at *p* < 0.05. NS—lack of statistical significance, NDM—nondiabetic control rats, T1DM—untreated type 1 diabetic rats, SIL50—type 1 diabetic rats treated with silymarin at a dose of 50 mg/kg, and SIL100—type 1 diabetic rats treated with silymarin at a dose of 100 mg/kg.

**Table 3:** Effect of silymarin on glycation and selected oxidative stress parameters in the serum and the lenses of type 1 diabetic rats.

Parameter/Group	NDM	T1DM	SIL50	SIL100
**Serum**				
MDA [nmol/mL]	11.06 ± 1.39 ^b^	33.94 ± 15.98 ^a^	30.11 ± 10.84 ^a^	28.70 ± 6.56 ^a^
AOPP [μmol/L]	23.46 ± 2.49 ^NS^	38.39 ± 9.59 ^NS^	38.58 ± 22.73 ^NS^	33.16 ± 11.20 ^NS^
AGEs [μg/mL]	6.59 ± 1.55 ^b^	9.12 ± 0.75 ^a^	6.89 ± 0.49 ^b^	7.49 ± 1.71 ^b^
**Lenses**				
MDA [nmol/g]	5.89 ± 0.27 ^b^	7.48 ± 0.94 ^a^	6.29 ± 0.66 ^b^	6.17 ± 0.95 ^b^
AOPP [nmol/mg of protein]	3.72 ± 0.41 ^b^	4.69 ± 0.94 ^a^	2.43 ± 0.50 ^c^	2.62 ± 0.51 ^c^
PCG [nmol/mg of protein]	0.020 ± 0.003 ^b^	0.027 ± 0.005 ^a^	0.027 ± 0.006 ^a^	0.020 ± 0.004 ^b^
G6PD [U/mg of protein]	0.040 ± 0.006 ^NS^	0.029 ± 0.011 ^NS^	0.035 ± 0.015 ^NS^	0.041 ± 0.015 ^NS^
GR [nmol/min/mg of protein]	0.51 ± 0.19 ^NS^	0.33 ± 0.10 ^NS^	0.43 ± 0.22 ^NS^	0.34 ± 0.10 ^NS^
Vitamin C [μg/g]	7.69 ± 0.14 ^a^	7.17 ± 0.06 ^b^	7.23 ± 0.09 ^b^	7.14 ± 0.11 ^b^
AGEs [μg/g]	7.47 ± 1.88 ^b^	10.83 ± 0.49 ^a^	11.01 ± 1.31 ^a^	9.91 ± 0.82 ^a^

Results in the table are presented as arithmetical means ± standard deviation (*n* = 8–9 for lenses assays and *n* = 7–9 for serological assays). Statistical significances were evaluated with one-way ANOVA followed by Fisher’s LSD post hoc test. The letters (a–c) in the superscripts indicate statistical significances. Values presented for each parameter in individual rows sharing at least one letter reveal no statistically significant differences at *p* < 0.05. NS—lack of statistical significance, NDM—nondiabetic control rats, T1DM—untreated type 1 diabetic rats, SIL50—type 1 diabetic rats treated with silymarin at a dose of 50 mg/kg, SIL100—type 1 diabetic rats treated with silymarin at a dose of 100 mg/kg, MDA—malondialdehyde, AOPP—advanced oxidation protein products, PCG—protein carbonyl groups, AGEs—advanced glycation end products, G6PD—glucose-6-phosphate dehydrogenase, and GR—glutathione reductase.

**Table 4:** Effect of silymarin on polyol pathway markers in the lenses of type 1 diabetic rats.

Parameter/Group	NDM	T1DM	SIL50	SIL100
Glucose [μmol/g]	0.90 ± 0.60 ^b^	4.63 ± 1.11 ^a^	4.98 ± 1.18 ^a^	5.09 ± 0.73 ^a^
AR [nmol/min/mg of protein]	0.086 ± 0.011 ^b^	0.107 ± 0.021 ^a^	0.109 ± 0.013 ^a^	0.123 ± 0.029 ^a^
Sorbitol [μmol/g]	1.23 ± 0.15 ^c^	30.62 ± 1.85 ^ab^	29.67 ± 2.85 ^b^	31.52 ± 1.66 ^a^
SDH [μU/mg of protein]	1.97 ± 0.46 ^c^	2.93 ± 0.87 ^b^	2.41 ± 0.56 ^bc^	3.81 ± 1.13 ^a^
Fructose [μmol/g]	0.076 ± 0.002 ^c^	0.135 ± 0.007 ^ab^	0.142 ± 0.008 ^a^	0.132 ± 0.015 ^b^

Results in the table are presented as arithmetical means ± standard deviation (*n* = 8–9). Statistical significances were evaluated with one-way ANOVA followed by Fisher’s LSD post hoc test. The letters (a–c) in the superscripts indicate statistical significances. Values presented for each parameter in individual rows sharing at least one letter reveal no statistically significant differences at *p* < 0.05. NDM—nondiabetic control rats, T1DM—untreated type 1 diabetic rats, SIL50—type 1 diabetic rats treated with silymarin at a dose of 50 mg/kg, SIL100—type 1 diabetic rats treated with silymarin at a dose of 100 mg/kg, AR—aldose reductase, and SDH—sorbitol dehydrogenase.

## Data Availability

Data are contained within the article.
